# Antimicrobial susceptibility testing of *Dermabacter hominis*

**DOI:** 10.1128/spectrum.01827-24

**Published:** 2024-11-15

**Authors:** Tim Kintzinger, Dennis Knaack, Sören Schubert, Uwe Groß, Robin Köck, Frieder Schaumburg

**Affiliations:** 1 Institute of Medical Microbiology, University Hospital Münster, Münster, Germany; 2 Competence Center Microbiology and Hygiene, St. Franziskus Hospital Münster, Münster, Germany; 3 Max von Pettenkofer Institute, Ludwig-Maximilians-University München, München, Germany; 4 Institute of Medical Microbiology and Virology, Universitätsmedizin Göttingen, Göttingen, Germany; 5 Hygiene and Environmental Medicine, Universitätsmedizin Essen, Essen, Germany; Icahn School of Medicine at Mount Sinai, New York, New York, USA

**Keywords:** *Dermabacter hominis*, antimicrobial susceptibility testing, broth microdilution, disk diffusion, breakpoints

## Abstract

*Dermabacter hominis,* a short gram-positive rod, is a part of the human skin flora, but can also cause infections (e.g., skin and soft tissue infections, bone and joint infections, abscesses, peritoneal dialysis-associated peritonitis, and bacteremia). Only limited data are available for antimicrobial resistance rates. Although CLSI does include coryneform genera in *Corynebacterium* spp. clinical breakpoints, they point out that only limited data are available on resistance rates. The aim of this study was to assess the minimal inhibitory concentration (MIC) of clinical isolates of *D. hominis* and to deduce breakpoints for disk diffusion. *D. hominis* (n = 30) from five laboratories in Germany were tested by broth microdilution and disk diffusion method. MICs were interpreted according to current clinical breakpoints for *Corynebacterium* spp. or pharmacokinetic–pharmacodynamic breakpoints (EUCAST). To deduce breakpoints for disk diffusion, MICs were correlated with inhibition zone diameters. All isolates were susceptible to vancomycin, rifampicin, and linezolid (100%, *n* = 30/30). Lower susceptibility rates were found for ampicillin (83%, *n* = 25/30) followed by ceftriaxone (37%, *n* = 11/30) and clindamycin (27%, *n* = 8/30). All isolates were resistant to benzylpenicillin and daptomycin. Good correlations between disk diffusion and MIC (suggested breakpoints for susceptibility in brackets) were found for ampicillin (S ≥ 10 mm), ceftriaxone (S ≥ 24 mm), clindamycin (S ≥ 19 mm), levofloxacin (I ≥ 24 mm), linezolid (S ≥ 29 mm), rifampicin (S ≥ 38 mm), and vancomycin (S ≥ 21 mm). Due to limited variances in both MIC values and inhibition zone diameters, no disk diffusion breakpoint could be deduced for gentamicin and benzylpenicillin in our dataset. *D. hominis* has favorable susceptibility rates for vancomycin, rifampicin, and linezolid and shows correlations between MIC and disk diffusion diameter for selected antimicrobial agents. Thus, the development of clinical breakpoints for disk diffusion appears feasible.

**IMPORTANCE:**

*Dermabacter hominis* can cause infections in humans (e.g., skin and soft tissue infections, bone and joint infections, abscesses, peritoneal dialysis-associated peritonitis, and bacteremia). Currently, only limited data are available regarding the resistance rates of this specific pathogen. Data for the easy accessible disk diffusion method are missing. We were able to provide additional data on resistance rates of clinical *D. hominis* isolates to common antimicrobial agents and correlate these with disk diffusion diameters to derive breakpoints to further improve the antimicrobial susceptibility testing for this specific pathogen. In addition to that, we created a current overview of resistance rates from the existing literature. Our data provide deeper insight into resistance rates and antimicrobial susceptibility testing of this specific pathogen.

## INTRODUCTION

*Dermabacter hominis* is a short gram-positive rod, which is a part of the normal skin flora of humans (1). In immunocompromised patients, or patients with significant comorbidities, it can cause infections (e.g., skin and soft tissue infections, bone and joint infections, abscesses, peritoneal dialysis-associated peritonitis, and bacteremia) (2–9). However, this occurs only in a small number of cases (10).

Antimicrobial susceptibility testing (AST) is a prerequisite for a targeted treatment. The gold standard is the broth microdilution (BMD) to measure the minimal inhibitory concentration (MIC). However, BMD is time-consuming and may not be established for all bacterial species in routine diagnostics. Currently, there are no validated clinical breakpoints available for AST of *D. hominis* using neither BMD nor disk diffusion (11). If species-related breakpoints are not available, the European Committee on Antimicrobial Susceptibility Testing (EUCAST) recommends using pharmacokinetic–pharmacodynamic (PK–PD), non-species-related breakpoints or epidemiological cut-off values (ECOFFs). PK–PD breakpoints are based on pharmacological and pharmacodynamical calculations but do not consider clinical outcome data. ECOFFs separate the wild-type phenotypes from isolates with acquired resistance mechanisms (12).

Currently, only limited antimicrobial susceptibility data of *D. hominis* using BMD are available. In addition, neither EUCAST nor Clinical and Laboratory Standards Institute (CLSI) breakpoints are available for the interpretation of disk diffusion. Therefore, the objectives of this study were to assess the susceptibility rates of clinical *D. hominis* isolates to commonly used antimicrobial agents using BMD and to deduce potential breakpoints for disk diffusion from MICs.

## MATERIALS AND METHODS

### Bacterial isolates

We prospectively collected 37 clinical *D. hominis* isolates from five routine laboratories in Germany (2021–2023). All isolates were identified by Matrix-assisted laser desorption/ionization time-of-flight mass spectrometry (MALDI-TOF MS; MALDI Biotyper® sirius one IVD System, Bruker, Bremen, Germany) using the MBT Compass IVD 4.2 database. The only inclusion criterion was confirmed species of *D. hominis* from colonization or infection. Duplicate isolates or isolates, which were unable to be suspended in sodium chloride (for BMD and disk diffusion), were excluded. A sample size calculation was not done, because the exact resistance rates for most antibiotics were unknown. A convenience sample of ≥30 isolates was deemed appropriate for the study objectives (13).

### Phylogenetic analysis

**Fig 1:**
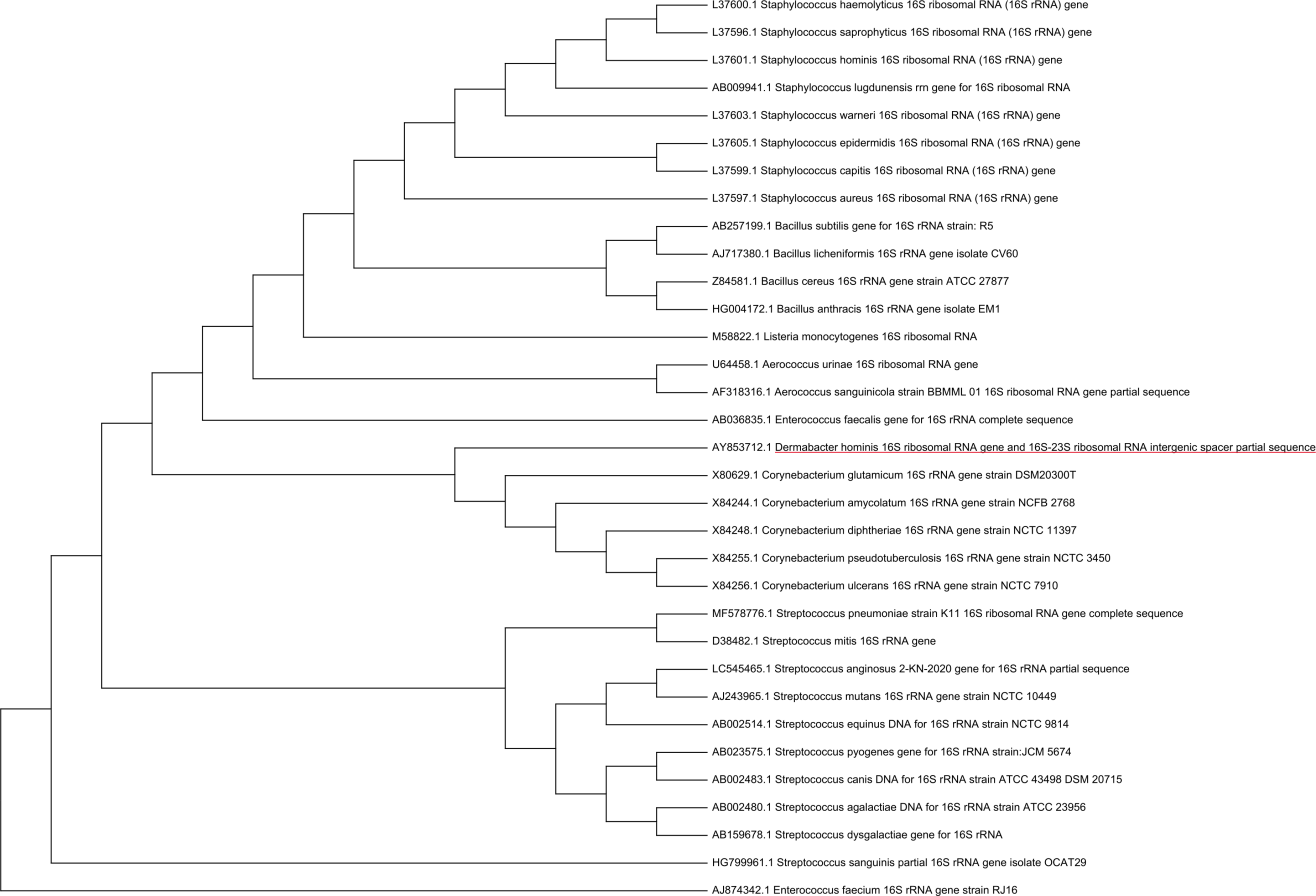
Phylogenetic (neighbor-joining) tree based on 16S RNA gene sequences of *Dermabacter hominis* and closely related species using MEGA software.

A phylogenetic tree was built to identify those bacterial species other than *D. hominis* for which EUCAST breakpoints are available and which are closely related to *D. hominis*. For that purpose, a neighbor-joining tree was created based on the 16S RNA gene sequences of *D. hominis* (Accession number: AY853712.1) using MEGA software (Version 11.0.13, Fig. 1). Sequences from closely related species were retrieved from GenBank (14).

### Antimicrobial susceptibility testing

For BMD, we used commercial 96-well plates (MICRONAUT-S GP varia complete MIC, MERLIN Diagnostica, Berlin, Germany). Bacterial isolates were dissolved in sodium chloride, adjusted to 2 × 10^5^–8 × 10^5^ colony forming units (CFU)/mL, transferred into Mueller–Hinton Fastidious broth (MH-F broth, Liofilchem, Roseto degli Abruzzi, Italy) and plates were inoculated following the manufacturer‘s instruction. Plates were incubated at 35 ± 1°C in ambient air for a total of 40–44 h (11) following ISO 20776-1 (15) and were read visually according to EUCAST guidelines (16). The incubation period was extended to 40–44 h, as many isolates showed insufficient growth after 16–20 h. The purity of the solution and correct inoculum (5 × 10^5^ CFU/ml, range: 2 × 10^5^–8 × 10^5^ CFU/ml) were verified by culture of the inoculated broth on Columbia blood agar (BD, Heidelberg, Germany) at 35 ± 1°C in ambient air.

The MICs were interpreted either with clinical breakpoints for *Corynebacterium* spp., or PK–PD breakpoints if clinical breakpoints for the tested antibiotics were not available (11). MIC of daptomycin was interpreted using a cut-off value of R > 1 mg/L (7).

Disk diffusion was done following EUCAST recommendations for the testing of *Corynebacterium* spp (11). We used Mueller–Hinton Fastidious Agar plates (BD) and antimicrobial disks (Oxoid, Wesel, Germany) with concentrations as recommended by EUCAST (11). The isolates were incubated at 35±1°C, 5% CO_2_ for 18 ± 2 h (11). BMD and disk diffusion were performed in biological triplicate for each tested isolate with independent inocula preparation from a fresh overnight culture. We used the median when reporting MIC for individual isolates.

*Staphylococcus aureus* ATCC 29213, *Streptococcus pneumoniae* ATCC 49619, and *Escherichia coli* ATCC 25922 were used as quality control (QC) strains, and corresponding MIC values were always within the QC range according to EUCAST (17).

### Comparison with recently published data

We searched for recently published data using the search terms “*Dermabacter hominis* OR coryneform bacteria AND susceptibility” in PubMed. All publications on *D. hominis* that reported MIC values independent of the applied guideline (CLSI, EUCAST) were included. Publications were excluded if authors did not respond or did not provide MIC raw data. MIC raw data were interpreted according to current EUCAST breakpoints (11).

### Statistical analysis

The MIC, which inhibits the growth of 50% (MIC_50_) and 90% (MIC_90_) of the total number of isolates, was calculated for each antimicrobial agent. Furthermore, MIC ranges for BMD were reported for each antimicrobial agent. Correlations between MIC and inhibition zone diameter of disk diffusion were assessed by stacked bar charts (18).

All calculations and figures were created with Microsoft Excel 2016.

## RESULTS

In total, 37 isolates were eligible and seven had to be excluded due to the inability to be dissolved in sodium chloride (which is requisite to perform AST). Thus, the final dataset included 30 isolates. These isolates were of human origin and derived from superficial or deep (wound) swabs (*n* = 8/30), urine (*n* = 8/30), blood cultures (*n* = 6/30), or other specimens (*n* = 8/30). The median age was 67.5 years (26–90 years); the majority of patients were male (79%, *n* = 22/28). Data on the age and sex of two patients were not available. Further information regarding the clinical significance of the tested isolates was not available.

**Fig 2:**
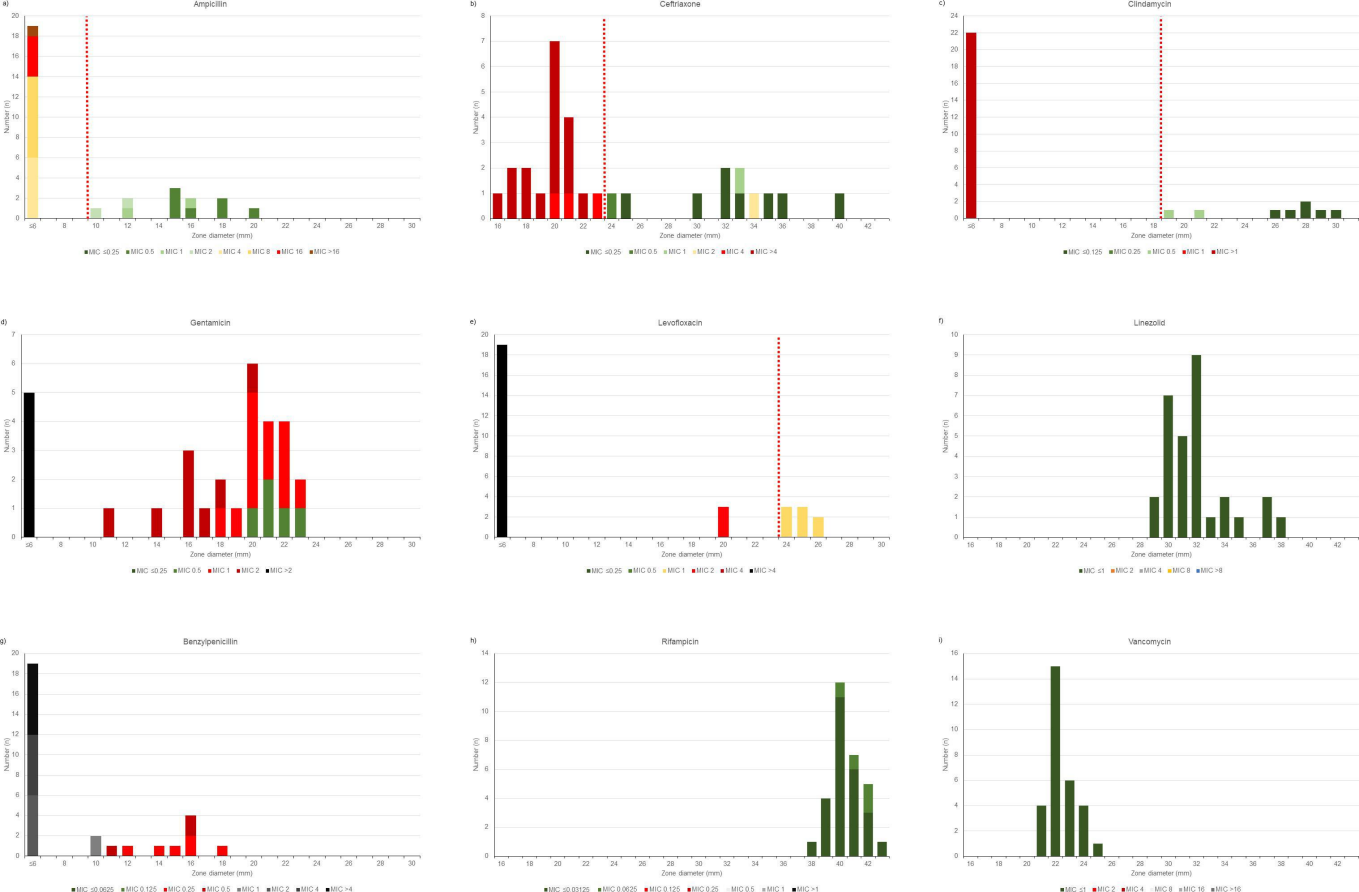
Distribution of minimal inhibitory concentrations (MICs) of *D. hominis. D. hominis* (*n* = 30) was tested by broth microdilution (BMD) and disk diffusion. MICs (staked bars) were plotted against disk diffusion diameters (horizontal axis) for ampicillin (**A**), ceftriaxone (**B**), clindamycin (**C**), gentamicin (**D**), levofloxacin (**E**), linezolid (**F**), benzylpenicillin (**G**), rifampicin (**H**), and vancomycin (**I**). Suggested values for disk diffusion breakpoints are indicated by red-dashed lines.

**Fig 3:**
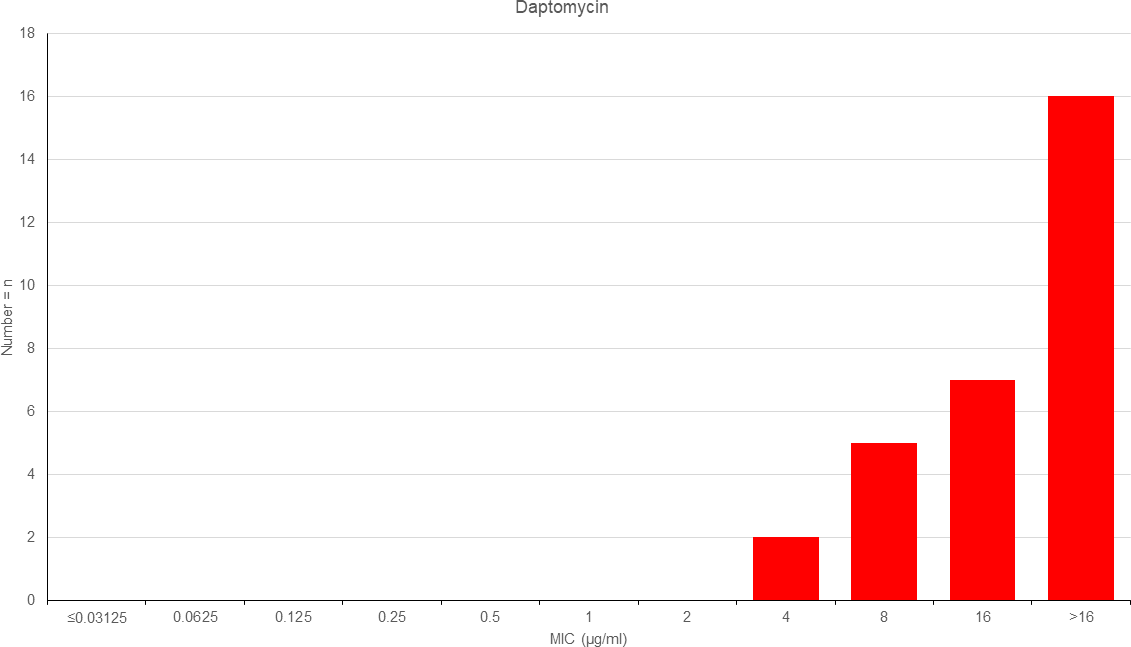
Distribution of MICs of *D. hominis* to daptomycin. *D. hominis* (*n* = 30) was tested by BMD. The number of isolates (vertical axis) is plotted against MIC (horizontal axis).

Using BMD, all isolates were susceptible to vancomycin, rifampicin, and linezolid (100%, *n* = 30/30, Table 1). Lower proportions of isolates were susceptible to ampicillin (83%, *n* = 25/30) followed by ceftriaxone (37%, *n* = 11/30) and clindamycin (27%, *n* = 8/30). All isolates were resistant to benzylpenicillin and daptomycin (Table 1). Visual inspection of MIC values shows a Gaussian normal distribution of *D. hominis* for ceftriaxone, gentamicin, linezolid, rifampicin, and vancomycin (Fig. 2). The distribution of MIC for daptomycin suggests that the peak MIC is >16 mg/L (Fig. 3).

**TABLE 1:** Minimal inhibitory concentrations (MICs) and susceptibility rates of *Dermabacter hominis* from Germany, 2021–2023 (*n* = 30)

Antimicrobial agent	MIC_50_ (mg/L)	MIC_90_ (mg/L)	Range (min–max)	Susceptibility, % (*n*)	S (mg/L)	R (mg/L)	Reference for breakpoints
Ampicillin	4	16	0.5–>16	83 (25)	≤2	>8	(11)
Ceftriaxone	>4	>4	≤0.25–>4	37 (11)	≤1	>2	(11)
Clindamycin	>1	>1	≤0.125–>1	27 (8)	≤0.5	>0.5	(11)
Daptomycin	>16	>16	4–>16	0 (0)	≤1	>1	(7)
Fosfomycin	16	32	≤8–64	3 (1)	≤8	>8	(11)
Gentamicin	1	>2	0.5–>2	17 (5)	≤0.5	>0.5	(11)
Levofloxacin	>4	>4	1–>4	27 (8)	≤0.5	>0.5	(11)
Linezolid	≤1	≤1	Not applicable	100 (30)	≤2	>2	(11)
Benzylpenicillin	2	>4	0.25–>4	0 (0)	≤0.125	>0.125	(11)
Rifampicin	≤0.03	≤0.03	≤0.03–0.06	100 (30)	≤0.06	>0.06	(11)
Vancomycin	≤1	≤1	Not applicable	100 (30)	≤2	>2	(11)

All isolates were subsequently tested by disk diffusion to assess if inhibition zone diameters correspond to MIC. For that purpose, stacked bar charts of MIC values and inhibition zone diameters were created (Fig. 2). Good correlations between disk diffusion and MICs (suggested breakpoints for susceptibility in brackets) were found for ampicillin (S ≥ 10 mm), ceftriaxone (S ≥ 24 mm), clindamycin (S ≥ 19 mm), levofloxacin (I ≥ 24 mm), linezolid (S ≥ 29 mm), rifampicin (S ≥ 38 mm), and vancomycin (S ≥ 21 mm, Fig. 2).

Due to limited variances in MIC values (e.g., the absence of MIC values covering both resistant and susceptible isolates) and/or in inhibition zone diameters, no disk diffusion breakpoint could be deduced for benzylpenicillin, rifampicin, vancomycin, and daptomycin in our dataset (Fig. 2 and 3). For gentamicin, the deduction of disk diffusion breakpoints appears challenging due to an overlap of three MIC dilution steps (0.5–2 mg/L, Fig. 2) covering both susceptible and resistant isolates at one inhibition zone diameter (20 mm).

The compilation of MIC values from published literature (7, 19) and our study confirmed that all isolates were susceptible to vancomycin and linezolid (100%, *n* = 64/64, Table 2). Rifampicin showed a slight decrease in susceptibility rates (92%, *n* = 59/64, Table 2). Lower proportions of isolates were susceptible to ampicillin (89%, *n* = 39/44), followed by ceftriaxone (62%, *n* = 31/50) and clindamycin (34%, *n* = 22/64). Benzylpenicillin (14%, *n* = 6/44), gentamicin (11%, *n* = 7/64), and fosfomycin (4%, *n* = 2/50) showed poor susceptibility (Table 2). Only one isolate was susceptible to daptomycin (2%, *n* = 1/44, Table 2). MIC values for levofloxacin were missing in the published literature. We showed a low proportion of susceptible isolates to levofloxacin (27%, *n* = 8/30, Table 1).

**TABLE 2:** Compilation of MICs and susceptibility rates of *D. hominis* from the literature

		Susceptibility % (*n*/total)				Reference for breakpoints
		This study	Fernández-Natal et al. (7)	Troxler et al. (19)	Total	
AST test conditions	Method	BMD in sodium chloride	Gradient diffusion	BMD in H medium	NA^*b*^	NA
	Incubation time [h]	40–44	24–48	22	NA	NA
Antimicrobial agent	Ampicillin	83 (25/30)	100 (14/14)	–^*a*^	89 (39/44)	(11)
	Ceftriaxone	37 (11/30)	–	100 (20/20)	62 (31/50)	(11)
	Clindamycin	27 (8/30)	21 (3/14)	55 (11/20)	34 (22/64)	(11)
	Daptomycin	0 (0/30)	7 (1/14)	–	2 (1/44)	(7)
	Fosfomycin	3 (1/30)	–	5 (1/20)	4 (2/50)	(11)
	Gentamicin	17 (5/30)	14 (2/14)	0 (0/20)	11 (7/64)	(11)
	Levofloxacin	27 (8/30)	–	–	27 (8/30)	(11)
	Linezolid	100 (30/30)	100 (14/14)	–	100 (44/44)	(11)
	Benzylpenicillin	0 (0/30)	43 (6/14)	–	14 (6/44)	(11)
	Rifampicin	100 (30/30)	100 (14/14)	75 (15 (20)	92 (59/64)	(11)
	Vancomycin	100 (30/30)	100 (14/14)	100 (20/20)	100 (64/64)	(11)

^*a*^ –, no data available.

^*b*^ NA, not applicable.

## DISCUSSION

We performed BMD and disk diffusion on 30 clinical *D. hominis* isolates from five German laboratories. *D. hominis* showed favorable resistance rates to vancomycin, rifampicin, and linezolid as mentioned in the literature before (7, 19). Based on good correlations between disk diffusion and MICs, we were able to deduce disk diffusion breakpoints for ampicillin, ceftriaxone, clindamycin, levofloxacin, linezolid, rifampicin, and vancomycin. Which of these antimicrobial agents are best for the treatment of *D. hominis* infections needs to be assessed in clinical trials. Further steps need to be taken for the development of clinical breakpoints, including the calculation of an ECOFF, collecting PK–PD data from *in vivo* and *in vitro* studies, implementing modeling processes, such as Monte Carlo simulation and correlating to clinical outcome data (20). For the calculation of an ECOFF, EUCAST states that the aggregated MIC distribution must contain at least 100 MIC values in the putative wild-type distribution (21). Therefore, the published number of *D. hominis* with MIC values still does not meet the minimum requirements by EUCAST. Thus, our complied data from three studies (incl. ours) should be interpreted with caution.

We were able to demonstrate a high resistance rate to daptomycin (100%, *n* = 30/30, Table 1), which is consistent with the results previously reported in the literature (7).

The daptomycin resistance is most likely due to the ability of *D. hominis* to modulate ether-linked lipids in the presence of daptomycin but must be further investigated (22).

Our study has limitations: First, we used commercial plates and not self-prepared stocks as recommended by ISO 20776–1. However, several studies showed a good correlation between the BMD reference method by EUCAST and commercial micronaut systems for other pathogens (23–26). Therefore, we rate this limitation as minor. Second, a greater number of isolates from other regions and sources (e.g., animals and colonization) needs to be tested to achieve higher reliability and create definite zone diameter breakpoints as required by EUCAST (27). Third, PK–PD breakpoints for fosfomycin are only applicable for oral treatment of uncomplicated UTI infections, which are not reported for *D. hominis* so far. Fourth, the compilation of MICs from the literature (Table 2) needs to be interpreted with caution as the tests were not standardized and, therefore, most likely not comparable.

### Conclusion

*D. hominis* has favorable susceptibility rates for vancomycin, rifampicin, and linezolid and shows correlations between MIC and disk diffusion diameter for selected antimicrobial agents. The development of clinical breakpoints for BMD and for disk diffusion, therefore, appears feasible.

## Data Availability

The data sets used and analyzed during the current study are available from the corresponding author upon reasonable request.

## References

[1] Jones D, Collins MD. 1988. Taxonomic studies on some human cutaneous coryneform bacteria: description of Dermabacter hominis gen.nov., sp.nov. FEMS Microbiol Lett 51:51–55. doi:10.1016/0378-1097(88)90228-5

[2] Bavbek M, Caner H, Arslan H, Demirhan B, Tunçbilek S, Altinörs N. 1998. Cerebral Dermabacter hominis abscess. Infection 26:181–183. doi:10.1007/BF02771848 9646113

[3] Gómez-Garcés JL, Oteo J, García G, Aracil B, Alós JI, Funke G. 2001. Bacteremia by Dermabacter hominis, a rare pathogen. J Clin Microbiol 39:2356–2357. doi:10.1128/JCM.39.6.2356-2357.2001 11376092 PMC88146

[4] Radtke A, Bergh K, Øien CM, Bevanger LS. 2001. Peritoneal dialysis-associated peritonitis caused by Dermabacter hominis. J Clin Microbiol 39:3420–3421. doi:10.1128/JCM.39.9.3420-3421.2001 11526195 PMC88363

[5] Van Bosterhaut B, Boucquey P, Janssens M, Wauters G, Delmée M. 2002. Chronic osteomyelitis due to Actinomyces neuii subspecies neuii and Dermabacter hominis. Eur J Clin Microbiol Infect Dis 21:486–487. doi:10.1007/s10096-002-0747-8 12111611

[6] Babay HA, Kambal AM. 2004. Isolation of coryneform bacteria from blood cultures of patients at a University Hospital in Saudi Arabia. Saudi Med J 25:1073–1079. 15322601

[7] Fernández-Natal I, Sáez-Nieto JA, Medina-Pascual MJ, Albersmeier A, Valdezate S, Guerra-Laso JM, Rodríguez H, Marrodán T, Parras T, Tauch A, Soriano F. 2013. Dermabacter hominis: a usually daptomycin-resistant Gram-Positive organism infrequently isolated from human clinical samples. New Microbes New Infect 1:35–40. doi:10.1002/2052-2975.31 25356327 PMC4184692

[8] Bertona E, De Paulis AN, Gutiérrez MA, Santa María V, Vay CA, Predari SC. 2016. Unusually infected sebaceous cyst by Dermabacter hominis. Rev Arg Microbiol 48:303–307. doi:10.1016/j.ram.2016.09.003 27773466

[9] Larrondo J, Porte L, Gosch M, Cabrera R, Weitzel T. 2017. Trichobacteriosis axillaris caused by Dermabacter hominis. J Eur Acad Dermatol Venereol 31:e267–e268. doi:10.1111/jdv.14082 27976432

[10] Schaub C, Dräger S, Hinic V, Bassetti S, Frei R, Osthoff M. 2020. Relevance of Dermabacter hominis isolated from clinical samples, 2012-2016: a retrospective case series. Diagn Microbiol Infect Dis 98:115118. doi:10.1016/j.diagmicrobio.2020.115118 32683204

[11] EUCAST. 2023. The European Committee on antimicrobial susceptibility testing. Breakpoint tables for interpretation of MICs and zone diameters. Version 13.1, 2023. Available from: https://www.eucast.org/fileadmin/src/media/PDFs/EUCAST_files/Breakpoint_tables/v_13.1_Breakpoint_Tables.pdf

[12] Kahlmeter G, Turnidge J. 2022. How to: ECOFFs-the why, the how, and the don’ts of EUCAST epidemiological cutoff values. Clin Microbiol Infect 28:952–954. doi:10.1016/j.cmi.2022.02.024 35218980

[13] CLSI. 2022. Clinical and laboratory standards institute (CLSI) M39: analysis and presentation of cumulative antimicrobial susceptibility test data. 5th edition

[14] GenBank Database, National Library of Medicine. 2023. Available from: https://www.ncbi.nlm.nih.gov/genbank/. Retrieved 18 Oct 2023.

[15] Standardization IOf. 2019. ISO 20776-1:2019 Susceptibility testing of infectious agents and evaluation of performance of antimicrobial susceptibility test devices — Part 1: Broth micro-dilution reference method for testing the in vitro activity of antimicrobial agents against rapidly growing aerobic bacteria involved in infectious diseases

[16] EUCAST. 2022. Broth microdilution - EUCAST reading guide v 4.0. Available from: https://www.eucast.org/fileadmin/src/media/PDFs/EUCAST_files/Disk_test_documents/2022_manuals/Reading_guide_BMD_v_4.0_2022.pdf. Retrieved 16 Oct 2023.

[17] EUCAST. 2023. The European Committee on Antimicrobial Susceptibility Testing.Routine and extended internal quality control for MIC determination and disk diffusion as recommended by EUCAST. Available from: https://www.eucast.org/fileadmin/src/media/PDFs/EUCAST_files/QC/v_13.2_EUCAST_QC_tables_routine_and_extended_QC.pdf

[18] EUCAST. Establishing disk diffusion zone diameter breakpoints. Available from: https://www.eucast.org/fileadmin/src/media/PDFs/EUCAST_files/Disk_criteria/EUCAST_zdd_breakpoints_C.pdf

[19] Troxler R, Funke G, Von Graevenitz A, Stock I. 2001. Natural antibiotic susceptibility of recently established coryneform bacteria. Eur J Clin Microbiol Infect Dis 20:315–323. doi:10.1007/s100960100503 11453591

[20] EUCAST. 2021. Harmonisation of breakpoints for existing antimicrobia agents EUCAST SOP 2.4. Available from: https://www.eucast.org/fileadmin/src/media/PDFs/EUCAST_files/EUCAST_SOPs/2021/EUCAST_SOP_2.4_Setting_breakpoints_existing_agents_20211202.pdflast

[21] EUCAST. 2021. Standard operating procedure, MIC distributions and the setting of epidemiological cut-off (ECOFF) values EUCAST SOP 10.2, 2 December 2021. Available from: https://www.eucast.org/fileadmin/src/media/PDFs/EUCAST_files/EUCAST_SOPs/2021/EUCAST_SOP_10.2_MIC_distributions_and_epidemiological_cut-off_value__ECOFF__setting_20211202.pdf

[22] Valero-Guillén PL, Fernández-Natal I, Marrodán-Ciordia T, Tauch A, Soriano F. 2016. Ether-linked lipids of Dermabacter hominis, a human skin actinobacterium. Chem Phys Lipids 196:24–32. doi:10.1016/j.chemphyslip.2016.02.002 26867985

[23] Matuschek E, Åhman J, Webster C, Kahlmeter G. 2018. Antimicrobial susceptibility testing of colistin - evaluation of seven commercial MIC products against standard broth microdilution for Escherichia coli, Klebsiella pneumoniae, Pseudomonas aeruginosa, and Acinetobacter spp. Clin Microbiol Infect 24:865–870. doi:10.1016/j.cmi.2017.11.020 29221995

[24] Wattal C, Goel N, Oberoi JK, Datta S, Raveendran R. 2019. Performance of three commercial assays for colistin susceptibility in clinical isolates and Mcr-1 carrying reference strain. Indian J Med Microbiol 37:488–495. doi:10.4103/ijmm.IJMM_20_92 32436869

[25] Pfennigwerth N, Kaminski A, Korte-Berwanger M, Pfeifer Y, Simon M, Werner G, Jantsch J, Marlinghaus L, Gatermann SG. 2019. Evaluation of six commercial products for colistin susceptibility testing in Enterobacterales. Clin Microbiol Infect 25:1385–1389. doi:10.1016/j.cmi.2019.03.017 30928563

[26] Cordovana M, Ambretti S. 2020. Antibiotic susceptibility testing of anaerobic bacteria by broth microdilution method using the MICRONAUT-S Anaerobes MIC plates. Anaerobe 63:102217. doi:10.1016/j.anaerobe.2020.102217 32461082

[27] EUCAST. 2022. Standard operating procedure, procedure for establishing zone diameter breakpoints and quality control citeria for new antimicrobial agents EUCAST SOP 9.3. Available from: https://www.eucast.org/fileadmin/src/media/PDFs/EUCAST_files/EUCAST_SOPs/2022/EUCAST_SOP_9.2_Disk_diffusion_breakpoints_and_QC_ranges_final_20200721.pdf

